# Excimer laser coronary atherectomy prior to paclitaxel-coated balloon angioplasty for de novo coronary artery lesions

**DOI:** 10.1007/s10103-020-03019-w

**Published:** 2020-04-18

**Authors:** Takashi Shibui, Takaaki Tsuchiyama, Shinichiro Masuda, Sho Nagamine

**Affiliations:** Department of Cardiology, Tokyo Metropolitan Hiroo Hospital, 2-34-10, Ebisu, Shibuya-ku, Tokyo, 150-0013 Japan

**Keywords:** Laser coronary atherectomy, Drug-coated balloon, Coronary artery disease, Angioplasty

## Abstract

This study aimed to evaluate the efficacy and safety of excimer laser coronary atherectomy (ELCA) prior to paclitaxel-coated balloon angioplasty for de novo coronary artery lesions. This retrospective observational study analyzed 118 eligible patients with de novo coronary artery disease whose only percutaneous coronary intervention was a drug-coated balloon angioplasty (i.e., no subsequent stent placement). Data related to our primary outcomes of interest—incidence of major adverse cardiovascular and cerebral events (MACCE), and incidence of procedural complications (bailout stenting and minor complications)—were collected and retrospectively analyzed. ELCA was used significantly more often in the cases of main branch and ostial lesions (i.e., of the circumflex, right coronary, or left anterior descending arteries, or high lateral branch), normally associated with poor treatment outcomes (55.6% vs. 14.3%, *p* < 0.0005). However, the two groups were not different in terms of cumulative incidence as estimated by the Kaplan–Meier method (log-rank test, *p* = 0.603) and a causal relationship between ELCA and MACCE was not identified (OR, 2.223; 95% CI, 0.614–8.047; *p* = 0.223). This study confirms the safety of ELCA prior to paclitaxel DCB angioplasty to treat de novo coronary artery lesions. While difficult-to-treat lesions were significantly more prevalent in the group treated by ELCA, the study revealed similar efficiency as conventional pre-dilation methods. Our findings provide grounds for a prospective randomized trial with consistent lesion and procedural characteristics to evaluate the potential benefits of combining paclitaxel DCB angioplasty following ELCA for de novo coronary artery lesions.

## Introduction

Drug-coated balloons (DCB) are semi-compliant angioplasty balloons covered with an anti-restenotic drug that is rapidly released locally into the vessel wall during the balloon contact [1]. The paclitaxel DCB received coverage under Japan’s national health insurance in January 2014. Paclitaxel, that coats the balloon surface, is absorbed into the vascular wall upon inflation, which acts to inhibit neointimal hyperplasia, allowing the device to prevent restenosis better than conventional balloons [2, 3]. While the drug-eluting stents are commonly used to treat coronary artery disease (CAD) [4], they are poorly suited for small vessel and ostial lesions, for which no standard treatment methods exist.

Since its first clinical application in 1983, excimer laser coronary atherectomy (ELCA) has been used for a variety of complex conditions including thrombus, in-stent restenosis, chronic total occlusion, non-crossable lesions, saphenous vein graft failure, and stent under-expansion [5]. In Japan, the technology was approved as an advanced medical therapy in 2008 and has been covered by the national health insurance since May 2012. Our department had employed ELCA in about 300 procedures since its first use in August 2013 till the end of 2018. DCBs are inserted into the target vessels after their preparation using a scoring device to ensure the lumen diameter is sufficiently wide. Combining ELCA with vessel scoring is theorized to achieve even greater luminal expansion [6]. This combined strategy has been evaluated in several pathological situations, including femoropopliteal in-stent restenosis (ISR) [7, 8], coronary ISR [9, 10], coronary aneurism [11], acute coronary syndrome (ACS) [12], and peripheral arterial diseases [13], with variable effects. While the efficacy for de novo CAD has not been studied, we hypothesized that adjuvant ELCA prior to DCB insertion may reduce the risk of lesion recurrence and adverse events. We conducted a pilot study, where we analyzed 5 patients that underwent ELCA prior to DCB angioplasty for de novo coronary artery lesions in our department from 2013 to 2017, to treat device-uncrossable and other difficult-to-treat lesions. Follow-up coronary angiography, performed in four of these patients, found no evidence of lesion recurrence or adverse events.

## Objectives

To address the lack of studies on this topic and based on the promising preliminary results obtained by combining paclitaxel DCB angioplasty following excimer laser irradiation for de novo coronary artery lesions, we decided to evaluate the efficacy and safety of this strategy performed in the Department of Cardiology of Tokyo Metropolitan Hiroo Hospital.

## Methods

### Patients

Between 2013 and 2018, a total of 1854 percutaneous coronary interventions (PCIs) were performed at Tokyo Metropolitan Hiroo Hospital and registered in the National Clinical Database (NCD). The present study was performed with the approval of the hospital ethics committee. All the patients provided a written informed consent to receive the ELCA treatment and have their data utilized for this study. Patients under 20 years of age at the time of surgery, or with an unprotected left main CAD, were excluded from the analysis. The indication for ELCA was decided by the operator based on the imaging studies, namely, angiography and intravascular ultrasound (IVUS), optical coherence tomography (OCT), or optical frequency domain imaging (OFDI). This retrospective observational study analyzed 118 eligible patients of de novo CAD who underwent DCB angioplasty as the only PCI (i.e., no subsequent stent placement).

### Case registration, group allocation

In total, 27 patients underwent adjuvant ELCA prior to DCB angioplasty (“ELCA”), while 91 underwent pretreatment by conventional balloon or scoring device (“conventional”). The decision to use ELCA was made by the operator when balloon inflation alone was inadequate to prepare the affected region: e.g., in the event of non-crossing, thrombotic, calcified, or otherwise problematic lesions.

### Catheter procedure

All patients were pretreated with loading doses of aspirin 200 mg and clopidogrel 300 mg or prasugrel 20 mg before the PCI. Anticoagulation during the PCI was achieved with intravenous unfractionated heparin boluses to maintain an activated clotting time ≧ 300 s. All ELCA procedures were conducted using the Spectranetics CVX-300 platform (Spectranetics, Colorado, CO, USA), consisting of an excimer laser generator (CVX-300) and pulsed xenon-chlorine laser catheters capable of delivering excimer energy (wavelength 308 nm, pulse length 185 ns) from 45 to 80 mJ/mm^2^ (fluence) at pulse repetition rates of 25 to 80 Hz. The operator decided to treat the patients, with concentric 0.9 to 1.7-mm excimer laser catheters, based on the angiographic and intracoronary imaging studies. We used the 0.9-mm excimer laser catheter for small vessel or bend lesions such as proximal LCX. For other lesions, a 1.4-mm catheter was used. The maximum fluence and repetition rate was determined by the operator and their distribution was as follows: 41% patients received 45 mJ/mm^2^ at 25 Hz, 14% patients received 55 mJ/mm^2^ at 35 Hz, 41% patients received 60 mJ/mm^2^ at 40 Hz, and 3.7% (only one patient) received 80 mJ/mm^2^ at 80 Hz. The guiding catheter was filled with saline before irradiation. The operator advanced the laser catheter at a speed of 0.5 mm/s while an assistant injected saline at 2–3 ml/s. After the first round, angiography and intracoronary imaging were repeated in order to determine whether to modify fluence and repetition rate for the next round. After ELCA, PCI was performed according to the standard procedure. For all the patients, DCBs were used except for 4 patients who required bailout stenting.

### Post-PCI management

All the patients were given 100-mg aspirin and 75-mg clopidogrel or 3.75-mg prasugrel daily after the PCI. Optimal medications such as β-blockers, angiotensin-converting enzyme inhibitors (ACE-I), angiotensin II receptor blockers (ARB), and statins were prescribed at the discretion of the attending doctors.

### Data collection/analysis

The following data were collected from the patients’ medical records: age, sex, physical findings, clinical laboratory findings, treatment types, clinical adverse events, and treatment outcomes. Data related to our primary outcomes of interest—incidence of major adverse cardiovascular and cerebral events (MACCEs), and incidence of procedural complications (bailout stenting and minor complications)—were collected and retrospectively analyzed. MACCEs were defined as target vessel revascularization (TVR) within 1 year of treatment (efficacy assessment), cardiac death, myocardial infarction, and stroke (safety assessment).

### Angiographic analysis

Coronary angiograms were obtained for angiographic analysis and reviewed by an experienced observer. Quantitative coronary angiography (QCA) was performed by means of CAAS 5.9 (Pie Medical, Maastricht, the Netherlands.)

### Statistical analysis

Means were compared between the two groups by unpaired *t* test; percentages were compared using either Pearson’s chi-square test or Fisher’s exact test. Survival curves were estimated using the Kaplan–Meier method and compared between the two groups using the log-rank test. Additionally, the inverse probability weighting (IPW) estimator was used to test for causality: namely, whether MACCEs were attributable to ELCA. SPSS Statistics version 17.0 (SPSS Inc., Chicago, IL, USA) was used for all the statistical testing, with a two-tailed significance level of *p* < 0.05.

## Results

Table 1 presents the characteristics of the patients analyzed. The ELCA and conventional groups were not different in terms of age, sex, or smoking rates (22~23%). While hypertension and dyslipidemia affected the two groups at similar rates, diabetes was more prevalent in the latter (54% vs. 33%, *p* = 0.061). More than 30% of each group had a history of myocardial infarction, and acute coronary syndrome affected both the groups at comparable rates (10~15%). Likewise, drug usage rates (i.e., of statins, beta blockers, and ACE-I/ARB) were not significantly different between the groups.

**Table 1:** Patient characteristics

Variable	ELCA (*n* = 27)	Conventional (*n* = 91)	*p* value
Age	66.47 ± 9.86	69.47 ± 11.66	0.207
Gender, male	24 (88.9%)	69 (75.8%)	0.145
Current smoker	6 (23.1%)	20 (22.2%)	0.927
Diabetes	9 (33.3%)	49 (53.8%)	0.061
Hypertension	20 (74.1%)	70 (77.8%)	0.795
Dyslipidemia	16 (59.3%)	62 (68.1%)	0.392
Statins	22 (81.5%)	69 (75.8%)	0.539
β blockers	13 (48.1%)	50 (54.9%)	0.534
ACE—Is/ARBs	13 (48.1%)	56 (61.5%)	0.215
Prior MI	9 (34.6%)	35 (38.5%)	0.721
ACS	4 (14.8%)	10 (11.0%)	0.735

Data are presented as number of cases and relative percentage of the group’s total in parentheses, except for admission age presented as mean ± standard deviation. ACE-Is, angiotensin converting enzyme inhibitors; ARBs, angiotensin receptor blockers; MI, myocardial infarction; ACS, acute coronary syndrome; ELCA, excimer laser coronary atherectomy

Table 2 provides an overview of the lesions treated and the procedure-related data. Bailout stenting was performed for a significantly greater percentage of patients in the ELCA than in the conventional group (11.1% vs. 1.1%, *p* = 0.036). Additionally, ELCA was used significantly more often in the cases of main branch and ostial lesions (i.e., of the circumflex, right coronary, or left anterior descending arteries, or high lateral branch), normally associated with poor treatment outcomes (55.6% vs. 14.3%, *p* < 0.0005). On the other hand, conventional angioplasty was employed significantly more often to treat small vessel lesions (i.e., in peripheral vessels or non-main branches; reference diameter ≤ 2.5 mm) (83.5% vs. 55.6%, *p* = 0.002). Imaging guidance was used in 100% of the ELCA cases and 97.8% of the conventional cases; OCT/OFDI was used significantly more often in the ELCA group (66.6% vs. 20.9%, *p* < 0.0005), while IVUS was used significantly more frequently in the conventional group (76.9% vs. 33.3%, *p* < 0.0005).

**Table 2:** Lesion and procedure characteristics

Variable	ELCA (*n* = 27)	Conventional (*n* = 91)	*p* value
Urgent PCI	4 (14.8%)	11 (12.1%)	0.745
Bailout stent	3 (11.1%)	1 (1.1%)	0.036
Minor complications	2 (7.4%)	12 (13.2%)	0.519
B2C lesion	21 (77.8%)	72 (79.1%)	1.000
Ostial lesion	16 (59.3%)	50 (54.9%)	0.692
Bifurcation lesion	19 (70.4%)	50 (54.9%)	0.153
Main branch ostial lesion*	15 (55.6%)	13 (14.3%)	< 0.0005
Small vessel (< = 2.5 mm)	15 (55.6%)	76 (83.5%)	0.002
DCB diameter (mean, mm)	2.69 ± 0.48	2.39 ± 0.44	0.003
Total DCB length (mm)	20.93 ± 7.29	21.44 ± 8.82	0.783
DCB inflation pressure (atm)	7.33 ± 2.14	7.45 ± 2.00	0.806
DCB inflation time (sec)	53.04 ± 11.75	53.73 ± 12.24	0.809
Imaging device			< 0.0005
Angio-guided	0 (0.0%)	2 (2.2%)	
IVUS	9 (33.3%)	70 (76.9%)	
OCT/OFDI	18 (66.6%)	19 (20.9%)	

Data are presented as number of cases and relative percentage of the group’s total in parentheses, except for total DCB length, DCB inflation pressure, and DCB inflation time presented as mean ± standard deviation. Minor complication means minor dissection (less than type B) or side branch occlusion or access site bleeding. B2C Lesion means the lesion of ACC/AHA classification type B2 or C

*Main branch ostial lesion means ostial lesion of left circumflex or right coronary artery or left anterior descending artery or high lateral branch in trifurcation lesions

PCI, percutaneous coronary intervention; DCB, drug-coated balloon; atm, atmosphere; IVUS, intravascular ultrasound; OCT, optical coherence imaging; OFDI, optical domain frequency imaging; ELCA, excimer laser coronary atherectomy

**Fig. 1:**
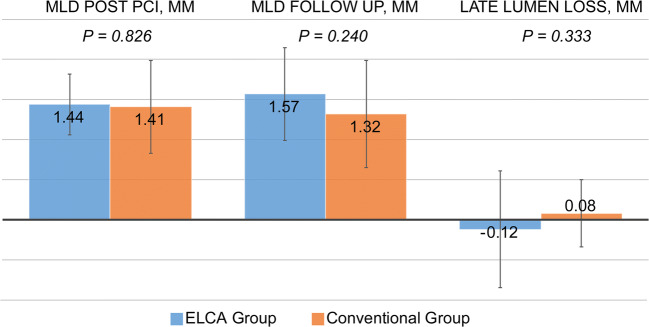
Minimum lumen diameter (MLD) post-PCI and at follow-up, and late lumen loss (LLL) in the conventional and ELCA group. Data are presented as mean ± standard deviation. PCI, percutaneous coronary intervention; ELCA, excimer laser coronary atherectomy

Laser catheters employed in the ELCA group usually had a diameter of 0.9 mm (*n* = 24), followed by 1.4 mm (*n* = 3), and 1.7 mm (*n* = 1), which was in agreement with our previous study [14] (Table 3). On an average, the maximum fluence was 53.7 mJ/mm^2^, the maximum frequency was 34.6 Hz, and the train count was 8.7. Quantitative coronary analysis evidenced a mean late lumen loss (LLL) of − 0.12 mm in the ELCA group, indicating a widening relative to the conventional group; however, this difference was not statistically significant (*p* = 0.333) (Table 3, Fig. 1).

**Table 3:** ELCA characteristics and quantitative coronary analysis

Variable	ELCA (*n* = 27)	Conventional (*n* = 91)	*p* value
ELCA			
Diameter 0.9 mm/1.4 mm/1.7 mm	24/3/1	–	
Maximum fluence (mJ)	53.7 ± 1.72	–	
Maximum frequency (Hz)	34.6 ± 2.20	–	
Total count	8.7 ± 1.7	–	
Post procedure			
MLD, mm	1.44 ± 0.38	1.41 ± 0.58	0.826
%Diameter stenosis	36.1 ± 3.37	33.9 ± 2.48	0.646
Follow-up			
MLD, mm	1.57 ± 0.77	1.32 ± 0.67	0.240
%Diameter stenosis	36.5 ± 6.04	38.3 ± 3.21	0.775
Late lumen loss, mm	−0.12 ± 0.73	0.08 ± 0.42	0.333

Data are presented as mean ± standard deviation, except for ELCA diameter presented as number of cases. ELCA, excimer laser coronary atherectomy; MLD, minimal lumen diameter

**Fig. 2:**
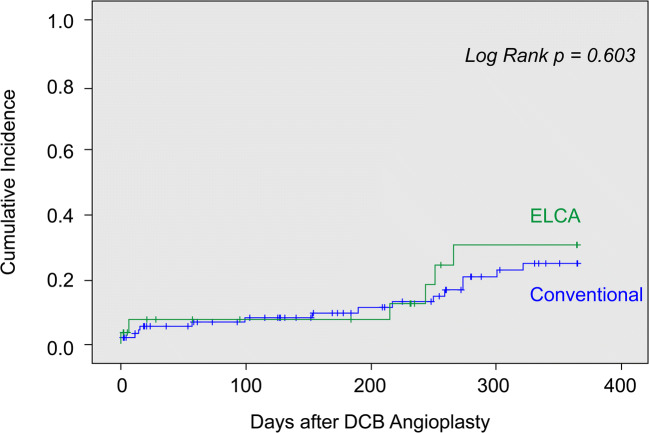
Major adverse cardiac and cerebrovascular events (MACCE) in the conventional and ELCA group. Inverse probability weighting (IPW) estimator was used to test for causality: namely, whether cumulative incidence of MACCEs were attributable to ELCA. PCI, percutaneous coronary intervention; ELCA, excimer laser coronary atherectomy

The primary outcome of interest, MACCE incidence, was 22.2% in the ELCA group and 17.6% in the conventional group. However, the two groups were not different in terms of cumulative incidence as estimated by the Kaplan–Meier method (log-rank test, *p* = 0.603) (Fig. 2). Additionally, the IPW estimator failed to support a causal relationship between the ELCA group and MACCE (OR, 2.223; 95% CI, 0.614–8.047; *p* = 0.223). The incidences of procedural complications, the second primary outcome, in the two groups were 18.5% (ELCA) and 14.3% (conventional), and this difference was not significant (*p =* 0.556).

## Discussion

In the recent years, ELCA has proved to be a promising strategy for the treatment of complex PCIs [15], such as in-stent restenosis [16] and chronic total occlusion [17]. ELCA was also safely and efficiently used for myocardial salvage in patients with ST-elevation myocardial infarction [18], for lead extraction of cardiac implantable devices [19], for acute coronary syndrome [20], and acute myocardial infarction [14]. The efficiency of ELCA could be associated with its ability to vaporize thrombi, to induce the “stunned platelets” phenomenon that reduces platelet aggregation [21], and to exert protective effects on endothelial cells [22]. In 2016, Hirose and his colleagues at Tokyo Medical and Dental University reported ELCA to effectively enhance the DCB inflation to treat in-stent restenosis, describing facilitation as its primary mechanism of action [6]. In this study, we sought to verify the safety of this technology and evaluate its efficiency in the treatment of de novo coronary artery lesions. Our results revealed no difference in the incidence of adverse events between the conventional and the ELCA group, demonstrating the safety of this procedure. The characteristics of the lesions treated were highly diverse, partly because the study was not a prospective randomized trial. Consequently, difficult-to-treat lesions (main branch ostial lesions) were significantly more common in the ELCA group than the conventional group. Due to this bias, we should consider that adjuvant ELCA led to comparable outcomes for main branch ostial and other difficult-to-treat lesions.

According to one review, bailout stenting was indicated in 7 ~ 28% of the DCB procedures following pre-dilation with conventional means to treat de novo coronary artery lesions, although this rate is highly variable [23]. Compared with these estimates, the bailout stenting rate of 11.1% in our ELCA group certainly does not seem high; rather, the 1.1% rate of our conventional group is unusually low. One reason for this may have been the DCB’s broad applicability to peripheral, non-main-branch, and other small vessel lesions. The perceived difficulty of bailout stenting in such situations may have led us to proceed to DCB inflation to apply paclitaxel to the vessel wall without adequate pre-dilation. Furthermore, while significant restenosis was observed in the follow-up coronary angiography in such cases, many of them were ineligible for revascularization due to a lack of angina symptoms or a lack of objective ischemia. Since ischemic symptoms are a necessary indication for TVR, these patients were ineligible for it, which may have negatively biased the efficacy calculated for the ELCA group, given its greater prevalence for main branch ostial lesions. This result supports the fact that the ELCA is a potentially effective strategy for difficult-to-treat lesions as previously reported for the cases showing severe calcification [24, 25].

## Conclusion

This observational study confirmed the safety of excimer laser irradiation prior to paclitaxel DCB angioplasty to treat de novo coronary artery lesions. While difficult-to-treat lesions were significantly more prevalent in the group treated by excimer laser irradiation, the study revealed similar efficiency as conventional pre-dilation methods. Therefore, our findings provide grounds for a prospective randomized trial with consistent lesion and procedural characteristics to evaluate the potential benefits of combining paclitaxel DCB angioplasty following excimer laser irradiation for de novo coronary artery lesions.

## Limitations

Since data used in this study were retrospective, selection of patients was not randomized, and difficult-to-treat lesions were significantly more common in the ELCA group than the conventional group; therefore, this bias could prevent the determination of a potentially beneficial effect of ELCA prior to paclitaxel DCB angioplasty in the treatment of de novo coronary artery lesions. An additional limitation was the small sample size at a single medical center.

## Data Availability

Data are not available as sharing was not approved by the Ethics Committee to be shared.
